# COVID-19 pandemic and total knee arthroplasty: the concept of a containment sheet—a frugal innovation

**DOI:** 10.1186/s42836-020-00042-8

**Published:** 2020-08-27

**Authors:** Vikram Indrajit Shah, Sachin Upadhyay, Kalpesh Shah, Vipin Singh

**Affiliations:** 1 Department of Knee and Hip Arthroplasty, Shalby Hospitals, Ahmedabad, Gujarat India; 2 Department of Orthopaedics, NSCB Medical College, Jabalpur, MP India; 3 Department of Trauma and Knee and Hip Arthroplasty, Shalby Hospitals Jabalpur, Jabalpur, Madhya Pradesh India

**Keywords:** Frugal innovation, COVID-19, TKA, Aerosols, WOMAC, VAS

## Abstract

**Background:**

The primary purpose of the present study was to assess whether use of proposed containment sheet (so called “a frugal innovation”) minimizes the aerosol and splatter dispersion during total knee arthroplasty (TKA).

**Material and method:**

A total of 32 patients with knee osteoarthritis who were scheduled to undergo primary and unilateral TKA reported during the COVID-19 pandemic were enrolled into this prospective single-institution cohort study. Demographic and epidemiological data, travel and contact history were collected. Eligible cohort was randomly assigned to a study (TKA using containment sheet) group and a control group (TKA without containment sheet). Radiological and functional outcomes before operation and at the final follow-up were assessed using Western Ontario and Mc-master Universities Osteoarthritis Index score (WOMAC) and the visual analog scale (VAS). The primary outcome was the postoperative effectiveness of containment sheet and face shield, defined as the numbers of countable macroscopic aerosols and/or splatters to naked eyes. The level of significance was set at *p* < 0.05 levels.

**Results:**

Present cohort was comprised of 14 men (43.75%) and 18 women (56.25%) with an average age of 65.45 ± 4.07 years (range, 62–75 years). There were no statistically significant differences with regard to baseline parameters and perioperative demographics. Functional outcomes for knee function at the last follow-up showed significant improvement in both the groups (*p* < 0.05). Face shield showed significant number of aerosols/splatters in control group. Highest number/concentration of aerosols/splatters was contained within the sheet.

**Conclusion:**

The proposed containment sheet can minimize the dispersion of aerosols and splatters generated during TKA and provide a safe healthcare environment in a cost-effective manner.

## Introduction

On 30 January 2020, World Health Organization declared unanticipated outbreak of COVID-19 as a Public Health Emergency of International Concern [1]. In India, on March 20, 2020 Ministry of Health and Family Welfare have issued advisory for hospitals and medical education institutions in the context of Corona virus pandemic [2]. The primary objective is to appropriately prepare the health infrastructure and use the existing resources judicially during a potential spike in COVID-19 patients, while also limiting exposure in healthy individuals.

Elective procedures may be considered to play a vital role in the health system’s revenue as it contributes a significant chunk in operating expenses of any multi-specialty health setup. Unanticipated cancellation or postponing of scheduled procedures at the eleventh-hour was a matter of serious concern. This arbitrary decision will certainly have monetary aftermath for these hospitals. Furthermore, these institutions will face increased daily expenditure due to increased paid leave and paid time-off for their staff, among other unexpected expenses. Furthermore, it is not clear when canceled procedures could be rescheduled, because the entire world including India is still working to flatten the curve of the pandemic. At the moment, there are no specific therapeutic strategies or vaccines for infection.

Knee osteoarthritis (KOA) in India is the second most common rheumatologic problem with a prevalence of 22% to 39%. The incidence of knee osteoarthritis in Indian population is approximately 15 % higher when compared to Western countries. This is attributed to genetic predisposition towards knee arthritis and socio-economic culture of sitting cross-legged and squatting. Our institute is a high-volume arthroplasty centre where approximately 500 arthroplasties were successfully executed per month. In view of this context, it is imperative that we should continue to operate on risk benefit ratio and at the same time retain adequate facilities to treat patients when we arrive at the summit of the curve. To the best of our knowledge, there is paucity of literature concerning the stringent necessities in the operative theatre for a patient suspected of COVID-19 scheduled for TKA. This led us to work out innovative responses in the form of a containment sheet, which is called a “frugal innovation”. The primary purpose of the present study was to assess whether the use of proposed containment sheet minimizes the aerosol and splatter dispersion during TKA and provides safety to healthcare team in the best possible way under current circumstances.

## Materials and methods

Majority of patients had pre-booked appointments for the surgery, and were currently stranded due to the countrywide lockdown. Travel back to their home was not feasible for these “highly symptomatic patients”, with flight and train services having been halted. Aware of this situation, our advisory committee decided to develop and pilot an algorithm to deal with these unprecedented circumstances.

**Fig. 1:**
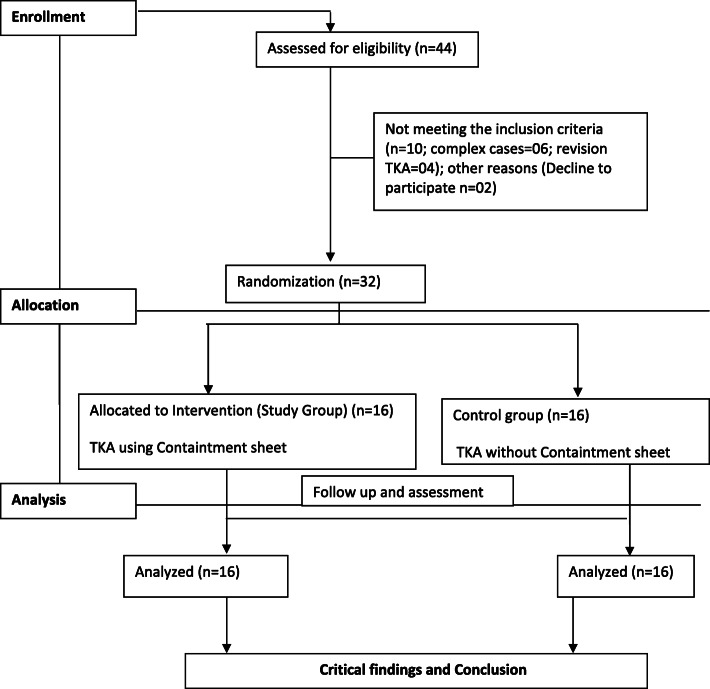
Consort flow chart

**Fig. 2:**
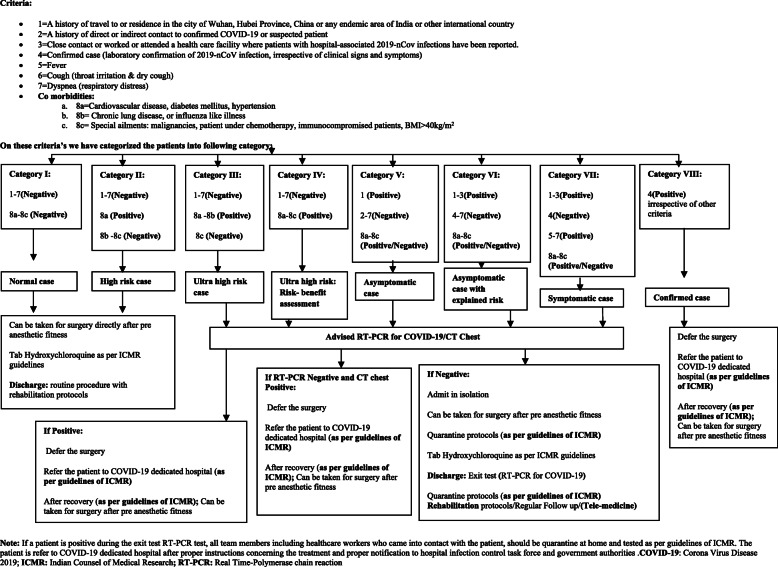
Algorithm: Institutional Criteria and Protocols for deciding management during COVID-19 outbreaks

After receiving approval from the institutional ethical committee, all patients with KOA scheduled to undergo primary and unilateral total knee arthroplasty reported to the institute during the period of global pandemic were enrolled in this prospective study. Post-septic knee arthritis, complex cases, bilateral cases, revision TKA and medically unfit patients were excluded from the study. Informed consent was obtained from all patients. A total of 44 patients were screened. As a result, 32 patients met the inclusion criteria while 12 patients were excluded. Consort flow chart for the study is shown in Fig. 1. The eligible cohort was randomly assigned to the study (TKA using containment sheet) and control groups (TKA without using containment sheet) by using a lottery system. Study was a 1:1 case control study. With social distance maintained, all demographic variables, including age, gender, body mass index (BMI), and comorbidities, were recorded. The preoperative knee alignment was assessed by scanogram and/or weight-bearing X-rays. Beside all routine arthritis-related information, a detailed epidemiologic history, including travel history, contact history and history of any constitutional systems *as per* guidelines from the Indian Council of Medical Research (ICMR), was noted. These patients were grouped into different categories according to our proposed algorithm (Fig. 2). Only RT-PCR-based assays were recommended. Conventional PCR, in-house real time PCR and antigen/antibody tests were not recommended for COVID-testing [2]. We have suggested RT-PCR test and chest CT scan as routine screening tests for category III patients onwards before being admitted to hospital for TKA and categorized as positive or negative. Preoperative screening for SARS-CoV-2 helped us implement mitigation strategies to avoid spread of infection among healthcare workers and patients. *As per* the algorithm, the patients were planned for the surgery. Before the surgery we have advised prophylactic use of tablet hydroxychloroquine against the COVID infection in both the patients and surgical team according to the recommendations issued by a national task force [2].

**Fig. 3:**
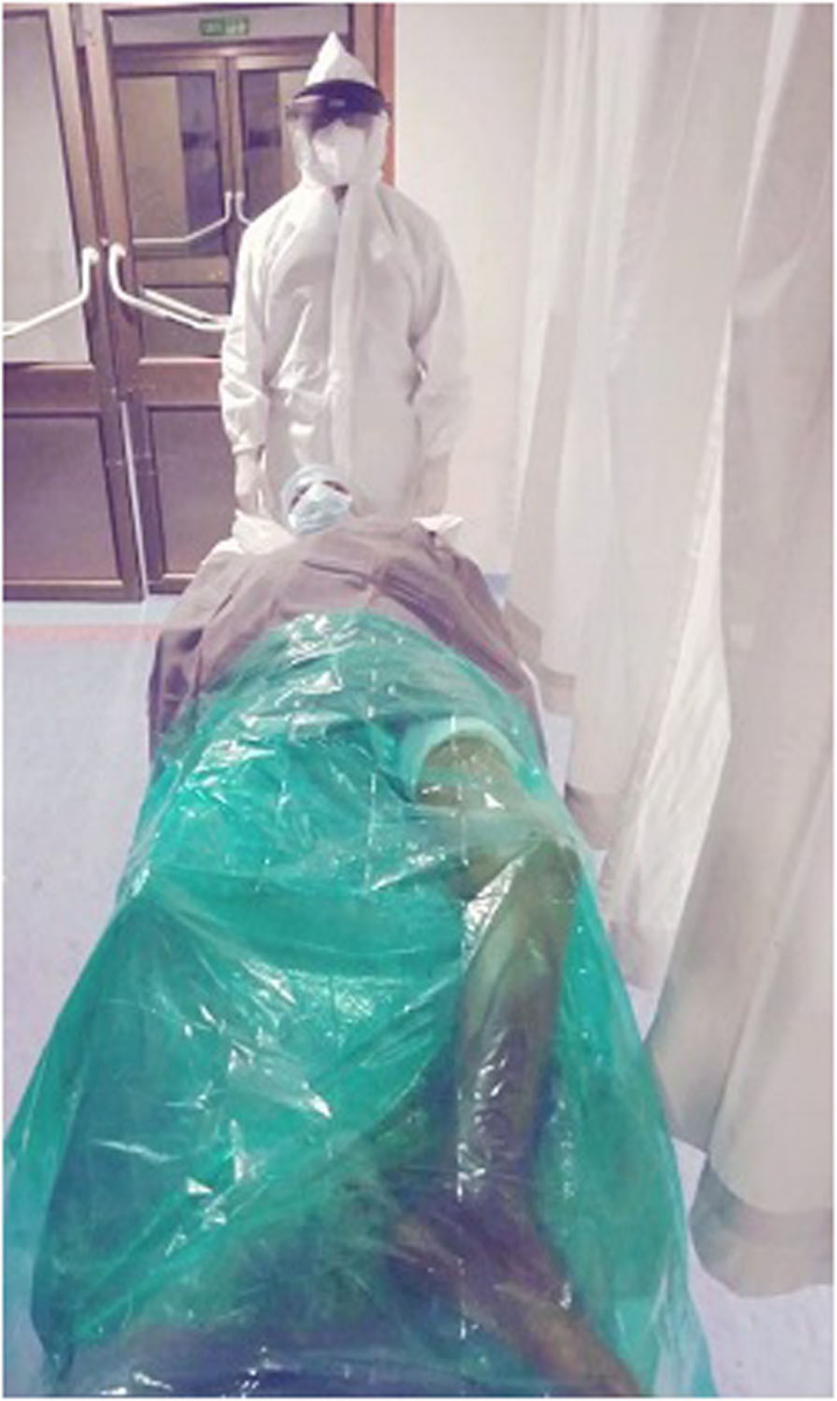
Patients wore a surgical mask and covered with plastic sheet during transfer to in OR and to the wards after the procedure

**Fig. 4:**
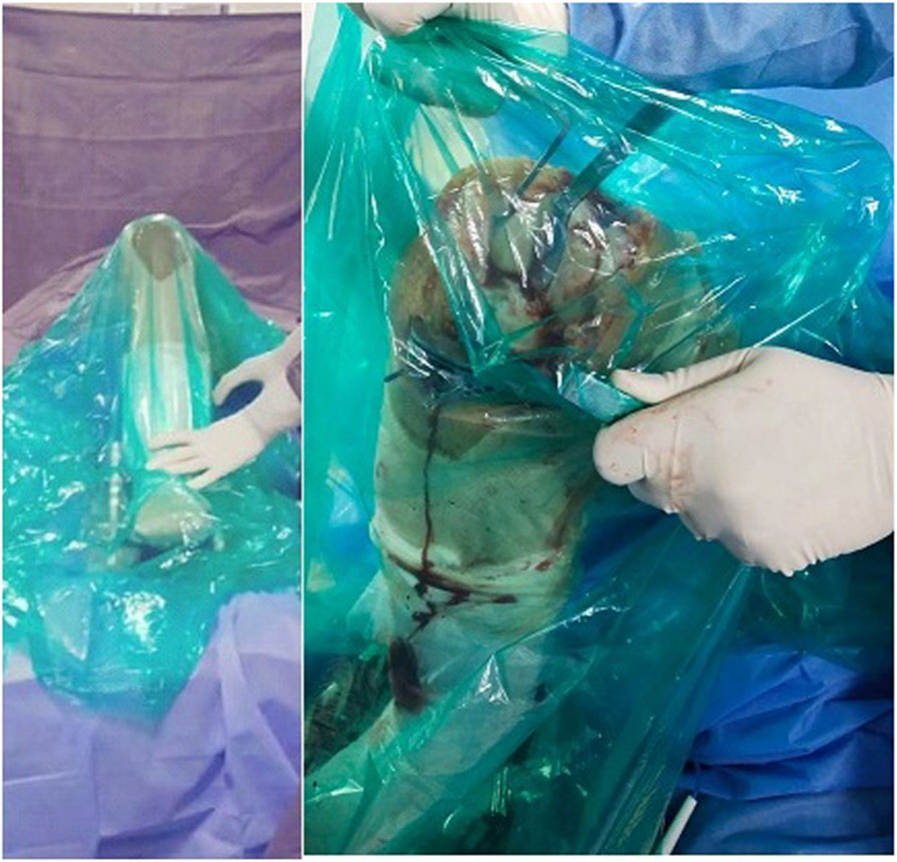
Use of containment sheet as a tent over the operative site

**Fig. 5:**
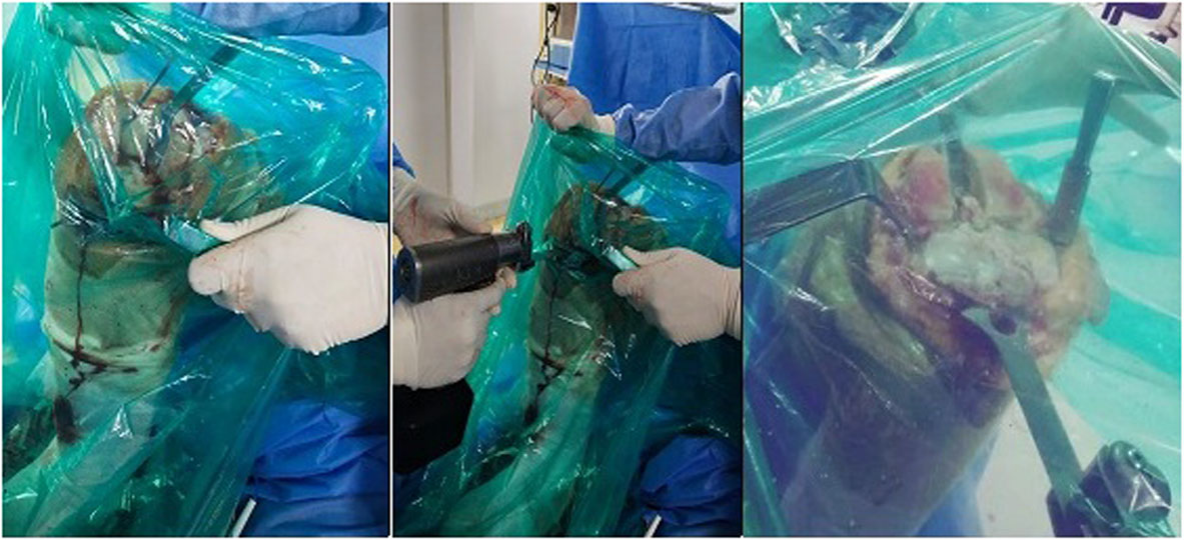
Phase two showing the routine tibial bony cuts under the containment sheet using power saw

**Fig. 6:**
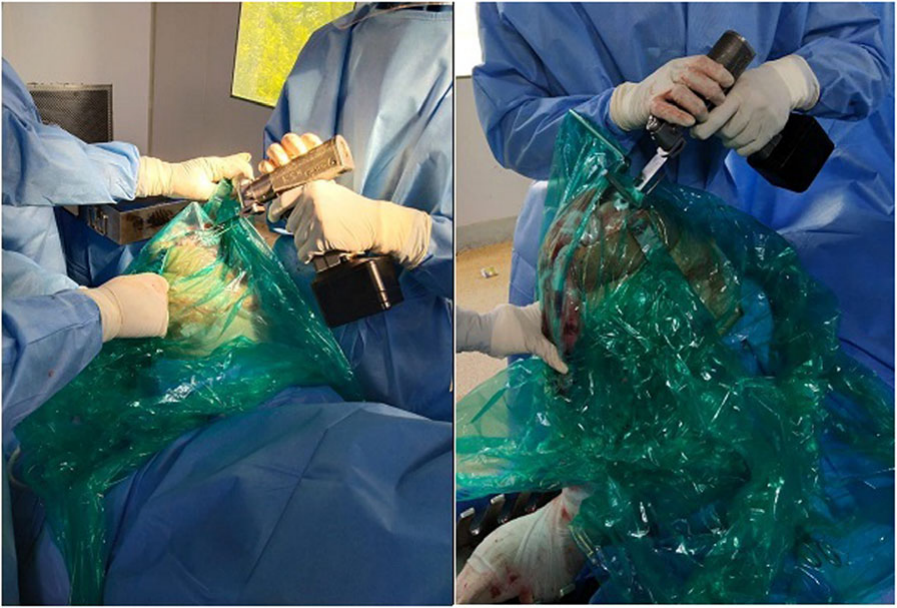
Phase three involved the femoral preparation and routine bony cuts under the containment sheet using power saw

All staff personnel were trained concerning the protective guidelines, donning/doffing and decontamination procedures. A separate area in the OT (operative theater) complex was earmarked for personal protective equipment (PPE) donning and doffing. During these extraordinary circumstances, we have modified the execution of our TKA to maximize patient and staff safety and to reduce the risk of contamination. All patients on the day of surgery were transferred directly to the designated operative room (OR) through an exclusive path and elevator by an attendant wearing standard PPE. All patients wore a surgical mask and were covered with plastic sheet during transfer to in OR and to the wards after the procedure (Fig. 3). Our protocols differed from the guidelines in that a containment sheet was applied over the operative area, which emphasizes full protection of the body suits and face shield from aerosols and other splashes generated during the procedure such as using power tools and suction and irrigation (Fig. 4). The rationale behind the use of such sheet was to minimize the dispersion of aerosols and droplets produced during the procedure. Containment sheet was a simple sterile transparent sheet, which was used as a tent over the surgical site as a closed compartment providing entry to the power saw, drill, electrocautry, suction cannula and irrigation tube through the controlled aperture made at appropriate site. This sheet was an assembly tailored to the area of the surgical site. Proper sealing of these sheets prevented both the ingress and/or egress of particles. To show the effectiveness of containment sheet against the dispersion of aerosols, the TKA was arbitrarily divided into different phases in terms of the different instruments and steps used. Phase one involved skin incision, medial para patellar arthrotomy and anterior dislocation of tibia. During this phase, the scalpel, electrocautery and, at the time of irrigation, suction system were used. Phase two included the tibial cut using power saw through the extramedullary jig. The saw was inserted through the aperture made in the containment sheet at the appropriate level using the cutting jig (Fig. 5). Phase three covered the femoral preparation and routine bony cuts under the containment sheet using power saw (Fig. 6). In phase four, the trial check and preparation for cementation were done. This involves the use of suction and irrigation system under the containment sheet through the apertures at appropriate place. In phase five, the prosthesis was finally implanted. Final phase involved suction and irrigation system and electrocautery for hemostasis. All operations were either performed or supervised by the senior author under standardized spinal anesthesia against strict protective guidelines set by the institute and were accomplished by using the pneumatic tourniquet system. All patients received prophylactic antibiotics and a single dose of intravenous tranexamic acid (10 mg/ kg body weight, maximum dose: 1 g) 15–20 min prior to skin incision. All surgeries were performed via a standard midline incision and medial parapatellar approach for arthrotomy with the patient assuming the supine position. Joint balancing was achieved by using standard cuts and appropriate soft tissue release. All had cemented posterior stabilized metal-backed PFC sigma fix bearing prosthesis. After irrigation, the joint was closed in layers without any drainage and no sutures were used superficially, with covering by an occlusive dressing. Staplers were used in cases where oozing was likely. The postoperative X-ray examination was done in the recovery room before the patient was shifted to the ward. All patients received standard chemoprophylaxis for VTE and were put on a standard protocol of rehabilitation.

For postoperative evaluation, all patients were followed up routinely through tele-medicine and advised to report only when oozing, wound healing problems, fever, respiratory distress, swelling or stiffness took place. For functional outcome evaluation, WOMAC and VAS were evaluated before operation and at the last follow-up. For radiographic evaluation, standard anteroposterior and lateral weight-bearing radiographs were analyzed for complication related to prosthesis before operation and at the last follow-up. For containment sheet evaluation, five custom-made sheets were chosen to collect the aerosols and the macroscopic aerosols/splatters, visible to naked eyes and/or captured by high resolution picture of the sheet, were counted. Face shield was evaluated with and without containment sheet. For statistical analysis, data were summarized, analyzed and expressed as mean ± SD. Any difference between independent means was analyzed by Student’s *t*-test with *p* < 0.05 as the level of statistical significance.

## Results

**Fig. 7:**
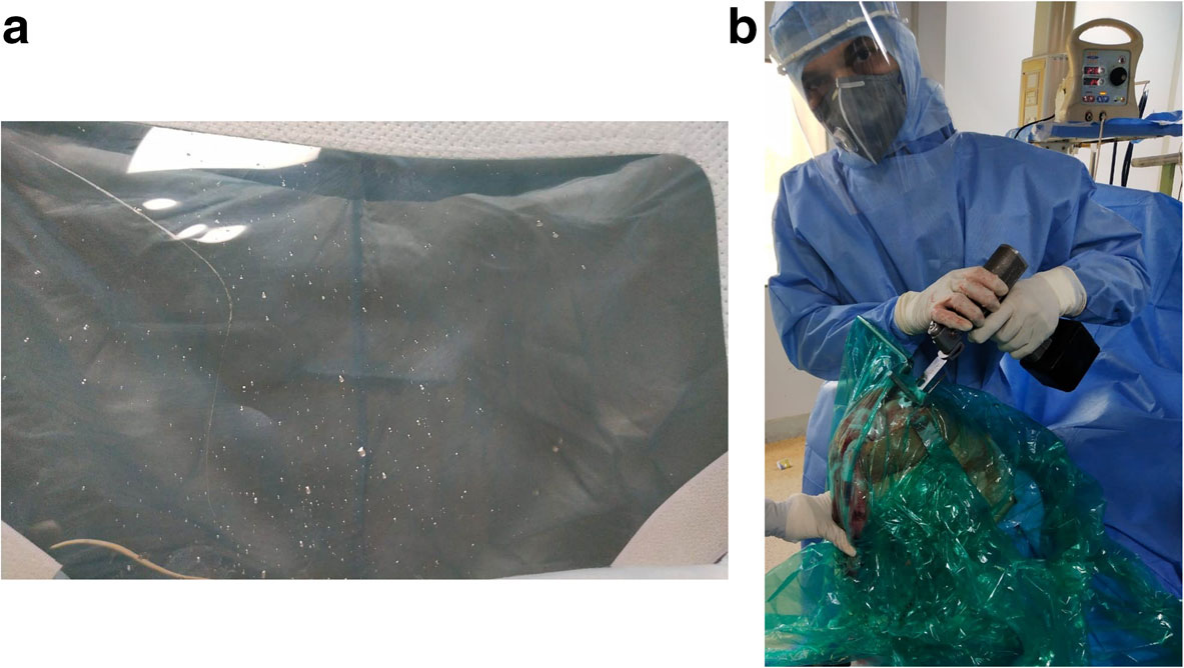
**a** Face shield showed significant number of aerosols/splatters in control group; **b** Study group had no aerosols

**Fig. 8:**
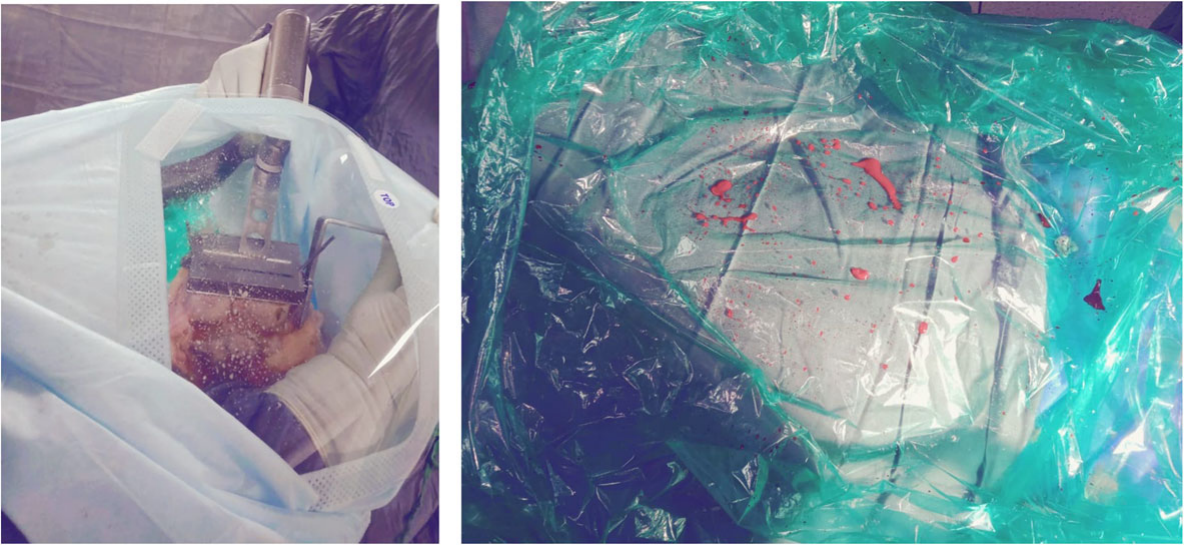
The inner aspect of containment sheet: concentration of aerosols/splatters was close to the surgical site and confined to the containment sheet

The present cohort was comprised of 14 men and 18 women, with an average age of 65.45 ± 4.07 years (range, 62–75 years). There were no statistically significant differences with regard to baseline parameters and perioperative demographics (Table 1). The average operative time and average duration of hospital stay between both groups were not significantly different (Table 2). Functional outcomes of knee at the last follow-up showed significant improvement (*p* < 0.05) in both the groups (Table 3). The containment sheet showed the highest number and/or concentration (when unable to count) of aerosols/splatters during phase two, three and four, followed by the sixth and fifth phases. During the phase one, the aerosol number and/or concentration (when counting was impossible) was almost insignificant. Face shield showed significant number of aerosols/splatters in control group (Table 4, Fig. 7). In study group, the concentration of aerosols/splatters was close to the surgical site and confined to the containment sheet (Fig. 8). No intraoperative complications were observed. The postoperative course was uneventful.

**Table 1:** Patients' demographic variables and baseline parameters

**Serial Number**	**Characteristics**	**Control (*n*** **= 16)**	**Study group (*n*** **= 16)**	***p*-value**
1.	Age (Years)	64.54 ± 5.22	66.19 ± 2.31	*p* = 0.2567
				*t* = 1.1562
2.	Gender	10 female (62.5%)	8 female (50%)	χ^2^ = 0.5079
		6 male (37.5%)	8 male (50%)	*p* = 0.476033
3.	Severity of disease (Kellgren and Lawrence system)	11 grade IV (68.75%)	13 grade IV (81.25%)	χ^2^ = 0.6667
		5 Grades III (31.25%)	3 Grade III (18.75%)	*p* = 0.414216
4.	Deformity (Femorotibial angle) (in degree) (Varus)	15.9 ± 1.03	16.3 ± 1.40	*p* = 0.3646
5.	Flexion angle (in degree)	85.8 ± 1.21	86.2 ± 1.01	*p* = 0.3182
6.	Co-morbidity (HTN, IHD, DM, ILI, COPD)	87.5% (*n* = 14)	93.75% (*n* = 15)	χ^2^ = 0.1829
				*p* = 0.668929
	**Category (proposed algorithm)**	**Control (*n*** **= 16)**	**Study group (*n*** **= 16)**	***p*-value**
7.	I	2 (12.5%)	1 (6.5%)	χ^2^ = 0.3678; *p* = 0.544197
	II	3 (18.75%)	2 (12.5%)	χ^2^ = 0.237; *p* = 0.626354
	III	3 (18.75%)	2 (12.5%)	χ^2^ = 0.237; *p* = 0 .626354
	IV	2 (12.5%)	3 (18.75%)	χ^2 ^= 0.237; *p* = 0.626354
	V	6 (37.5%)	8 (50%)	χ^2^ = 0.5079; *p* = 0 .476033
	VI	–	–	
	VII	–	–	
8.	WOMAC	52.70 ± 2.05	52.16 ± 1.88	*p* = 0.4435
9.	VAS	8.7 ± 1.02	8.5 ± 1.22	*p* = 0.6186

**Table 2:** Total WOMAC and VAS

Total WOMAC			
Group	Pre-operative (baseline)	Latest Follow up	***p***-value
**Control (*n*** **= 16)**	52.70 ± 2.05	24.3 ± 2.61	*p* < 0.0001
**Study group (*n*** **= 16)**	52.16 ± 1.88	23.7 ± 2.12	*p* < 0.0001
**VAS**			
**Group**			
**Control (*n*** **= 16)**	8.7 ± 1.02	2.98 ± 2.61	*p* < 0.0001
**Study group (*n*** **= 16)**	8.5 ± 1.22	3.25 ± 1.33	*p* < 0.0001

**Table 3:** Average operative time and hospital stay

Characteristics	Control (***n*** = 16)	Study group (***n*** = 16)	***p***-value
**Average Operative time (min)**	37.63 ± 3.22	39.69 ± 5.32	*p* = 0.1952
**Average hospital stay (days)**	2.81 ± 0.66	2.69 ± 0.48	*p* = 0.5608

**Table 4:** Face shield/Gown: Numbers of macroscopic aerosols/splatters/splashes

Characteristic	Control (***n*** = 16)	Study group (***n*** = 16)	***p***-value
**Face shield/Surgical gown**	631.25 ± 153.70	1.25 ± 1.00	*p* < 0.0001

## Discussion

The present research demonstrated that the use of containment sheets contained the aerosols/splatters and minimized their dispersion, thus reducing the risk of contamination.

During COVID-19 pandemic, we preferred to use the facility of multiple theatres in our hospital. The rationale behind this step is to allow a thorough cleaning of the operating room before the next case was introduced [3]. We sanitized and decontaminated all reusable articles on routine basis. All built-in instruments, such as ventilators, anesthetic monitors, and telephones, were covered with plastic paper to reduce the chance of contamination and to facilitate cleaning. All the instruments and surgical trays were properly autoclaved after each and every case. Contamination rate was correlated directly with the duration of exposure of the open instruments trays [4]. In view of present scenario, we does not prepare the surgery trolley until patient installation is completed to minimize the airborne contamination.

As there is substantial evidence that SARS-CoV-2 can survive up to days on surfaces made of metal, plastic or glass, proper cleaning and sanitation of room is mandatory [5]. Viricidal agents like dilute povidone iodine, 0.1% sodium hypochlorite solution, 62–71% ethanol or 0.5% hydrogen peroxide solution can be used as surface disinfectant with good efficacy [6]. Cleaners should wear full PPE while disinfecting surfaces.

Nowadays, almost all OT are fitted with HEPA (high efficiency particulate air) filters, which can efficiently remove particles ≥0.3 μm in diameter and thereby eliminate corona virus-loaded particles in aerosol form. Ideally, approximately 20 air changes/hour are required to dilute microbes generated in the theatre and to exclude ingress from adjoining areas [7]. Our hospital operating rooms are well equipped with laminar flow and HEPA capable of producing 40 air exchanges per hour. Negative pressure airborne infection isolation rooms (AIIR) are ideal in these challenging situations but owing to complex engineering procedures, cost and time involved, we have not advised to convert positive pressure room to AIIR. The contentment sheet that acts as a protective physical barrier to all particles and other precautionary steps discussed minimizes the aerosols/splatters generated and their dispersion during the procedure. Also the higher frequency of air exchanges reduces the viral load rapidly in the OR. If any patient becomes infected, laminar flow must be suspended.pr

All our surgeries were carried out under spinal anesthesia in strict accordance with institutional protocols. The patient wore a surgical mask throughout the procedure [8]. Also research has shown that mask over the source ensured better protection [9]. If sedation is required, supplemental oxygen may be given through nasal prongs underneath the surgical mask [10].

Before proceeding with the operative procedure, we have stored all necessary armantarium in the theatre and other unnecessary equipment was moved out so as to minimize the theatre traffic to avoid frequent door openings [11]. Studies reported that frequent door opening is proportional to the increased bacterial count [12]. Andersson *et al* reported that 7% of door openings were related to unpredicted issues concerning operative procedure and 26% to suboptimal theatre preparation and 27% to persons who hardly have any role in the procedure [13]. Increasing the number of staff personnel in the operating theater and their activities derange the airfield pattern and pose an enhanced risk of pathogen contamination and therefore influence the risk of infection [14]. In our procedures, we have reduced our team to four, including the anaesthetist. Furthermore, limiting the number of team members will help execute the physical distancing concept within the theatre and also decrease the demand for PPE by the theatre staff [3, 15]. Sadrizadeh S *et al* showed that increased staff personnel in theater was associated with a growing trend in the concentration of the bacteria-carrying particles and they recommended that airborne bacterial count should not exceed 10 CFU/m^3^ [16]. Aerosolized droplets is the most common mode of transmission of SARS-CoV-2, and therefore decreasing the air turbulence and the number of air particles, in turn, reduces pathogen transmission.

We have followed the concept of ‘time out’ or ‘surgical pause’ to eliminate any confusion among team members and avoided ‘wrong-patient’ or ‘wrong-site’ errors [17]. This concept also leads to ‘extended pause’, during which more protective measures concerning patient identity, team member safety and communication regarding the details of operative procedure are taken [18].

All the patients were given ‘prophylactic’ antibiotics immediately (average,15 min) before the incision was made so as to achieve better tissue and serum levels both at the start and at the end of the surgery [19]. A study confirmed the importance of administration of surgical antibiotics prophylaxis (SAP) and demonstrated that the administration within 30 min of incision ensured the lowest infection rate [20]. Re-dosing should be considered only in the cases of excessive intra-operative bleeding, a surgical procedure lasting for more than few hours or during prosthetic surgery.

**Fig. 9:**
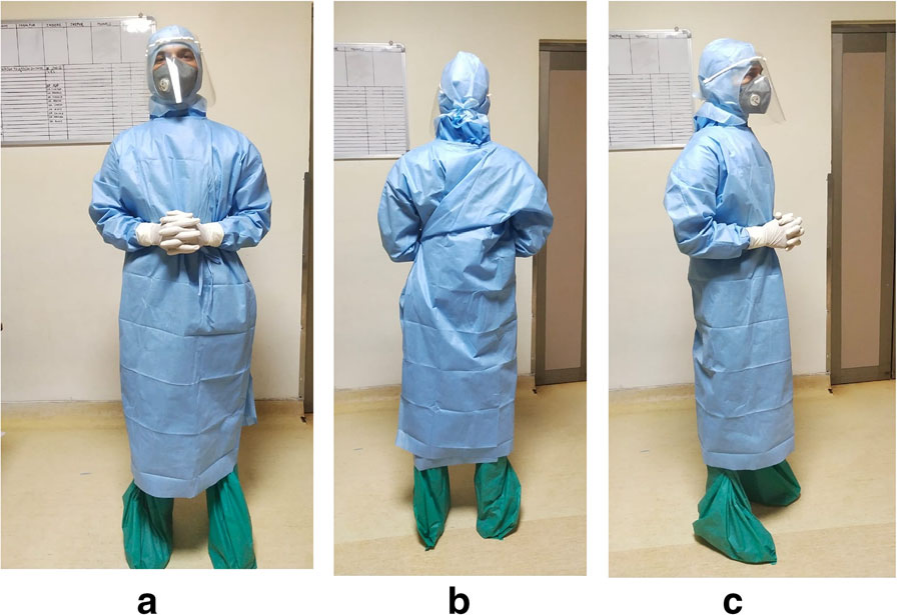
The protective gears: **a** Front; **b** Back; **c** Side

We have strictly followed the PPE guidelines set by our institution to minimize the potential exposure of staff to COVID/suspected COVID patients. Designated zones were prepared and earmarked in the operating theatre complex for donning and doffing of PPE. We have used N95 mask, face shield and neck strap in place of usual surgical helmets [1] (Fig. 9). Surgical helmets were designed to minimize the contamination risk and to protect from the splatters. Their efficiency in preventing respiratory droplet transmission is inadequate. Also, airflow can pull contaminated submicron-sized particles into the suit system, leading to contamination [21]. The authors do not recommend the use of these surgical helmets in the present scenario as the sterilization of these helmets between the cases was not feasible and also removing these helmets would be more difficult according to PPE protocols and further increases the risk of resuspesion of particles and contamination. During all procedures, we have used double indicator gloves assembly. This aims to recognize the fact that the gloves perforate easily intraoperatively and also it significantly reduces perforations of innermost gloves. Furthermore, it provides an effective second latex protective barrier, thus avoiding the possibility of potential inoculation of surgeon’s hand with contaminated materials [22]. Double gloving was associated with 50% reduction in infections and further reduce the risk of cross contamination [22]. The authors advocate change of outer gloves prior to incision, before touching the prosthesis and after cementation [23].

Our innovative “Zero-technique” maximally shortens surgical time to an average of 40 min. The average operating time in both groups did not differ significantly. This was surprising as the modified methodology in the study group did not require extra time. Increased operative time may enhance the risk of contamination and infection after TKA as it allows for prolonged exposure to airborne particles/microorganisms in the operating environment [24].

In TKA, surgical power tools, such as electrocautery, bone saws, drills, and reamers, were regularly used and these represent an important source for inhalable contaminated aerosols and splatters. So there should be a need of a cost-effective approach that helps in the containment of aerosols generated at the surgical site and minimizes the exposure of staff and essential equipment to aerosolized particles. This unprecedented phase encourages innovation and imagination, promoting us to use the containment sheet. The present study demonstrated that majority of the aerosols and splatters were contained within the containment sheet as shown by the particles deposited on the inner aspect of the sheet and also the evidence from the body suits and face shield. The face shield wore by the surgeon in the control group revealed higher concentration of aerosols/splatters than in the study group.

As another step to minimize the generation of aerosols, we tried to use the power saw at the lower frequency and to maintain the saw blade contact load to a maximum level. Pluim JME *et al* reported that higher saw frequency and low saw blade contact load led to higher production of aerosols [25]. Judicial use of an electrocautery (lower effective power, used with suction device to minimize the surgical smoke) and use of feeding tube as irrigation and suction cannula in place of pulse lavage system were others steps to minimize the production of aerosols during the procedure. Use of tourniquet and tranexamic acid prior to incision provides dry field and further minimizes the generation of blood-associated aerosols. Of note, all such procedures were performed using the containment sheet and, after each phase or when required, the sheets were carefully removed by using “folding-from-outer-to-inner” technique like a “paper roll” so as to keep the contents inside the fold. Otherwise it could have resuspended the particles.

There are limitations to the present study, including a small sample size and vague measurement of aerosols and splatters. Small sample size prevents the generalization of the finding and typically leads to Type-II errors. We have no idea about the actual diameter and total numbers of aerosols as we counted only the macroscopic splatters/aerosols visible to the naked eyes. Thus, data obtained from containment sheet and face shield may not be representative of operative environment. These statistics should be considered as a relative indication of aerosol concentrations and the dispersion during the surgery.

## Conclusion

The present critical analysis needs to be put in the perspective of the rapidly increasing cases of knee arthroplasties being performed every year. To the best of our knowledge, this is the first pilot report which highlights the protocol for executing TKA and use of containment sheet in the present situation. Our study has suggested that blood-associated splatters and inhalable aerosols were produced during TKA. The present research has demonstrated that the use of proposed containment sheet during surgery has the potential to contain and reduce the dispersion of aerosols and particles (both macroscopic and microscopic) in a cost-effective manner. In the absence of full protective gears, this type of frugal innovations provides a high-quality safe healthcare in the best possible way under the given circumstances and constraints. The aforementioned recommendations may be frugal, but they can mitigate risks to patients and staff. It may be worth future investigations to validate successful use of containment sheet during the surgery. We hope that this frugal innovation will help other hospitals worldwide in resuming TKA and other elective orthopaedic surgery in the present unexpected conditions.

## Data Availability

The data that support the findings of this study are available from [Shalby Hospitals India] but restrictions apply to the availability of these data, which were used under license for the current study, and so are not publicly available. Data are, however, available from the authors upon reasonable request and with permission of [Shalby Hospitals India].

## Abbreviations

TKA: Total knee arthroplasty
KOA: Knee Osteoarthritis
WOMAC: Western Ontario and Mc-master Universities Osteoarthritis Index score
VAS: Visual Analog Scale
ICMR: Indian Council of Medical Research
PPE: Personal Protective Equipment
